# A laminar augmented cascading flexible neural forest model for classification of cancer subtypes based on gene expression data

**DOI:** 10.1186/s12859-021-04391-2

**Published:** 2021-10-02

**Authors:** Lianxin Zhong, Qingfang Meng, Yuehui Chen, Lei Du, Peng Wu

**Affiliations:** 1 School of Information Science and Engineering, University of Jinan, Jinan, China; 2 Shandong Provincial Key laboratory of Network Based Intelligent Computing, Jinan, 250022 China

**Keywords:** Cancer subtype, Cascade forest, Classification, Deep learning, Ensemble methods

## Abstract

**Background:**

Correctly classifying the subtypes of cancer is of great significance for the in-depth study of cancer pathogenesis and the realization of personalized treatment for cancer patients. In recent years, classification of cancer subtypes using deep neural networks and gene expression data has gradually become a research hotspot. However, most classifiers may face overfitting and low classification accuracy when dealing with small sample size and high-dimensional biology data.

**Results:**

In this paper, a laminar augmented cascading flexible neural forest (LACFNForest) model was proposed to complete the classification of cancer subtypes. This model is a cascading flexible neural forest using deep flexible neural forest (DFNForest) as the base classifier. A hierarchical broadening ensemble method was proposed, which ensures the robustness of classification results and avoids the waste of model structure and function as much as possible. We also introduced an output judgment mechanism to each layer of the forest to reduce the computational complexity of the model. The deep neural forest was extended to the densely connected deep neural forest to improve the prediction results. The experiments on RNA-seq gene expression data showed that LACFNForest has better performance in the classification of cancer subtypes compared to the conventional methods.

**Conclusion:**

The LACFNForest model effectively improves the accuracy of cancer subtype classification with good robustness. It provides a new approach for the ensemble learning of classifiers in terms of structural design.

## Background

Cancer is an abnormal lesion formed when the organism, under the action of various carcinogenic factors, loses the normal regulation of the growth of local tissue cells at the genetic level, resulting in abnormal cell growth [1]. In the field of cancer research, the study of subtype classification is a very important task. Objective and accurate subtype classification of cancer can enable doctors to correctly understand the pathogenesis and primary location of cancer, which is of great significance to the study of cancer genesis [2]. Based on the different cancer sites and the different pathogenesis of the same type of cancer, the World Health Organization(WHO) has published guidelines for the classification of cancer and its subtypes, providing a global diagnostic standard for cancer and pathologists [3, 4].

With the completion of the Human Genome Project, large amounts of RNA sequence data need to be processed and analysed more rapidly [5]. Since then, high-throughput sequencing technologies have evolved, and many types of molecular biology data have been rapidly accumulated (mainly in terms of the characteristic dimension of the data, rather than the number of samples). Traditional analysis methods are difficult to meet the analysis requirements of high-dimensional data, so the use of bioinformatics to process genetic information at the molecular level has received increasing attention in recent years [6, 7]. Considering the comprehensiveness of gene expression data and its high correlation with cancer, it is highly feasible to use gene expression data to develop classification models for cancer subtypes [8].

The sequence of the entire human genome contains about 3 billion base pairs and tens of thousands of genes [9]. Gene expression data is essentially a high-dimensional data and its feature dimension is highly correlated with the number of genes [10]. Humans possess tens of thousands of genes, resulting in unprocessed gene expression data that typically has tens of thousands of features. On the one hand, the spatial structure of gene expression data is very complex and belongs to the high-dimensional space, so it is impossible to predict the distribution of data accurately [11]; on the other hand, gene expression data has high adaptability requirements for cancer subtype classification algorithms, so that conventional cancer subtype classification algorithms tend to fall into the local optimum in the process of finding the optimal one [12]. In addition, a large part of the gene expression profiles were obtained without considering labeling, which resulted in the gene expression data with labeling only accounting for a small part of the total data [13, 14].

In general, gene expression data are commonly characterized by high feature dimension, many missing values, small sample size, noise and redundant information [15]. All of these indicate that the classification of cancer subtypes based on gene expression data belongs to a class of biological information processing with high difficulty and complexity [16]. At this stage, the use of gene expression data to complete the classification of cancer subtypes is both valuable and challenging [17].

To solve these problems, Chen et al. [18] combined particle swarm and decision tree algorithms for simultaneous gene selection and cancer classification to identify genes that are highly correlated with specific tissues. Support vector machine (SVM) [19], as a bicategorical non-probabilistic algorithm, improved by plat scaling and other methods [20], has gradually become the most widely used method for cancer classification using gene expression data due to its excellent performance. Ramani et al. [21] proposed a cancer prediction framework for gene expression datasets, using the weights of rank the gene selection (RWFS) method to identify the features of various gene selection algorithms improves the classification accuracy of the model. Guo et al. [22] proposed the BCDForest framework based on Zhou et al. [23], which achieves better classification performance compared to the direct use of deep forest.

In this paper, we proposed a novel FNT-based deep neural network framework, the laminar augmented cascading flexible forest (LACFNForest), which not only breaks the dichotomous limitation of FNT, but also implements the forced deepening of DFNForest without introducing additional parameters. The main contributions are summarized as follows. (1) We proposed the idea of hierarchical broadening to enhance the diversity of the model and the number of classifiers layer by layer. This not only enhances the robustness of classification results, but also makes the whole model more interpretable, which provides a new idea for the structural design of classifier ensemble learning. (2) DFNForest was used as the base classifier of the model, which transforms the multi-classification problem into multiple dichotomous problems by M-ary method. The diversity and structural complexity of the classifiers were guaranteed by the way of cascading. (3) After obtaining the classification result of each forest layer, we assigned different weights to this result and introduce an output judgment mechanism to reduce the computational complexity of the model as much as possible while ensuring the accuracy of the classification result. (4) The structure of densely connected CNN was introduced into LACFNForest, which significantly improved the prediction accuracy of the model. The experimental results showed that when classifying the subtypes of breast invasive carcinoma (BRCA), lung cancer (LUNG) as well as glioblastoma multiforme (GBM), the classification accuracy of LACFNForest was significantly better than other current forms of classifiers.

## Results

### Datasets and parameters

The data used in this paper are RNA sequence gene expression data, which were obtained from The Cancer Genome Atlas (TCGA) [24]. TCGA is the largest open access cancer genome-wide database sponsored by the US government. In order to make better comparison with other classifier models, the experiments used the datasets of three kinds of cancers used in the literature [25]: breast invasive cancer (BRCA), glioblastoma multiforme (GBM) and lung cancer (LUNG). The labels of data samples are based on real clinical data of cancer patients provided by TCGA. There are four basic subtypes of BRCA: Basal-like, HER2-enriched, Luminal-A and Luminal-B and 514 valid data samples are available. There are three subtypes in LUNG: Bronchioid, Magnoid and Squamoid, with 275 effective samples. GBM has four basic subtypes: Classical, Mesenchymal, Neural and Proneural subtypes, and there are 164 valid samples available.

The Parameter of LACFNForest listed in Table 1 is mainly used by FNT which is the base classifier of our model. In the tree structure optimization algorithm using grammar guided genetic programming, we give the population size of 50, individual crossover and mutation probability were 0.4 and 0.01 respectively. When using particle swarm optimization to optimize the parameters of the FNT, we set the learning factors C1 and C2 to 2, and the maximum speed of particle movement to 2. The right side of the table shows some of the hyperparameters that are commonly used during CNN construction. While “Uncertain” indicates that the default value is unknown or that different settings are usually required for different tasks.

**Table 1:** Summarizes the hyper-parameters of deep neural networks (e.g., convolutional neural networks) and LACFNForest, where the default values used in our experiments are given

Parameter of LACFNForest	Value	Hyper-parameter of CNN	Value
Population size	50	No. hidden layers	Uncertain
Crossover probability	0.4	No. feature maps	Uncertain
Mutation probability	0.01	No. nodes in hidden layers	Uncertain
Level	4	Learning rate	Uncertain
C1	2.0	Kernal size	Uncertain
C2	2.0	Momentum	Uncertain
Vmax	2.0	L1/L2 weight regularization penalty	Uncertain

“Uncertain” indicates that the default values of the parameters are unknown or usually require different settings for different tasks. These hyperparameters do not exist in our model, or exist but can be determined automatically during the optimization process of our model

It is obvious that convolutional neural networks have many hyperparameters for which no definite values can be given. There are almost unlimited combinations of these parameters, and they determined the performance of CNN to a large extent. In different experiments, the values of these parameters are usually set based on the researcher’s experience. However, these hyperparameters do not exist in our model, or exist but can be determined automatically during the optimization process of our model.

The preprocessing steps are: outlier deletion, missing data imputation and normalization : if the gene expression data has more than 20% missing value in a patient, the patient data will be filtered; for the missing data, K-nearest neighbors is used to fill in; the normalization of cancer gene expression data is processed as follows:

1 $$\begin{aligned} {\tilde{f}}=\frac{f-E(f)}{\sqrt{{\text {Var}}(f)}} \end{aligned}$$

where *f* is the gene expression data feature, ${\tilde{f}}$ is the normalized gene expression feature, and *E*(*f*) and ${\text {Var}}(f)$ are the mean and variance of *f* [26].

### Comparison between the LACFNForest and other classifiers on the BRCA dataset

We used the proposed model on the BRCA dataset to test its classification performance. To demonstrate the superiority of LACFNForest in classification performance, we compared it with k-nearest neighbor (KNN), support vector machine (SVM), multilayer perception (MLP), random forest (RF), multi-granularity cascade forest (gcForest), and deep flexible neural forest (DFNForest), respectively. In the BRCA original dataset, each sample contains 4247 genes. The BRCA original data were used as the input for each classifier. And the experimental results are the average classification accuracies obtained by 10 experiments. To reduce the possibility of overfitting, in each experiment, the five-fold cross-validation was used to assess the overall accuracy of the different methods. The full dataset was randomly divided into 5 parts, each of the 5 parts was taken in turn as testing data (and the remaining as training data) in each of the 5 runs of a cross validation experiment. For each classifier, we compared the results in terms of average precision, recall, and F-1 score, and the results are shown in Table 2.

**Table 2:** Comparison of precision of KNN, SVM, MLP, RF, gcForest, DFNForest, and LACFNForest on the BRCA dataset

Method	KNN	SVM	RF	MLP	gcForest	DFNForest	LACFNForest
Precision	0.816	0.888	0.872	0.880	0.880	0.928	0.944
Recall	0.800	0.888	0.880	0.888	0.872	0.936	0.944
F-1 Score	0.808	0.888	0.876	0.884	0.876	0.932	0.944

**Fig. 1:**
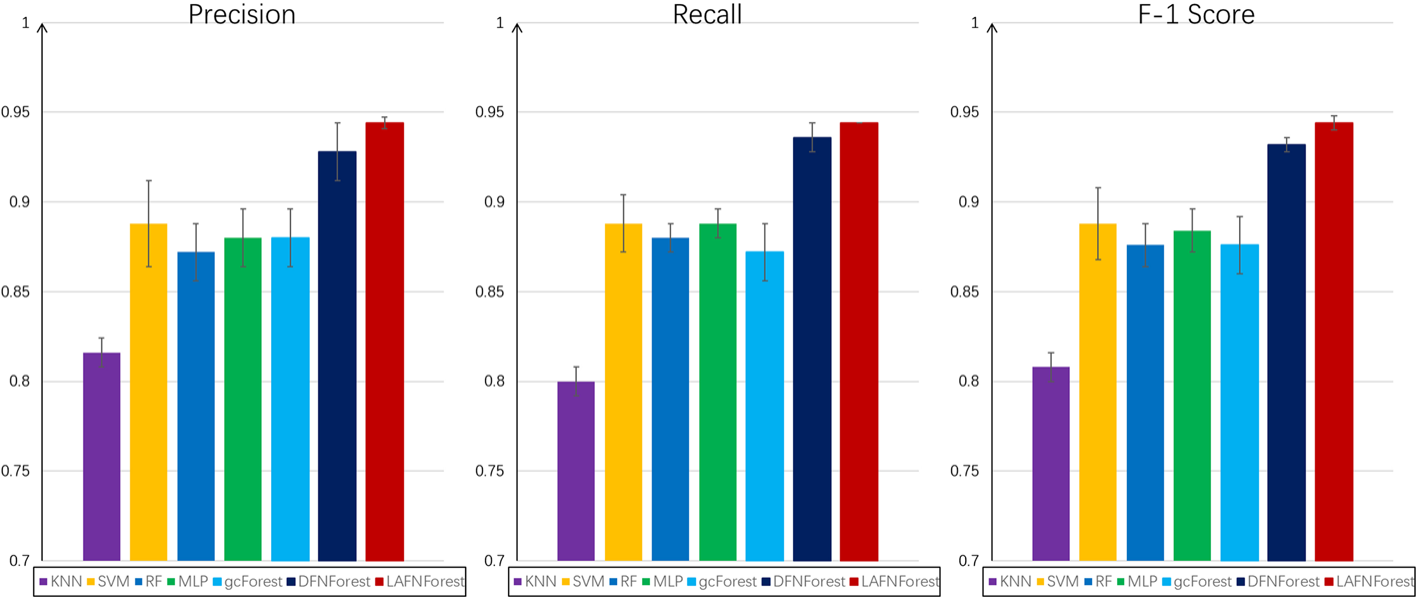
Comparison of the overall performance of multiple classifiers on the BRCA dataset: average precision, recall, F-1 score and their respective deviation values

In order to compare the classification results of different classifiers on the BRCA dataset more intuitively, we made the following comparison plots and give the deviation values of the experimental results (as shown in Fig. 1).

It can be seen that the LACFNForest model achieved 94.4% in classification accuracy when classifying cancer subtypes of BRCA. The classification performance is significantly better than other classifiers. The experimental results of the deviation values in Fig. 1 showed that our proposed model not only achieves higher classification accuracy, the classification results are also more stable, which shows that LACFNForest has good robustness in terms of its performance on data processing.

### Comparison between the LACFNForest and other classifiers on the GBM dataset

In processing the gene expression data, the same gene selection method was used in the GBM data set. The input for each classifier was the GBM original dataset, each sample contains 3398 genes. After obtaining the results of LACFNForest classification on GBM cancer subtypes, We compare it with KNN, SVM, MLP, RF, gcForest and DFNForest, respectively. For all the comparative experiments in this paper, the kNN method used k = 3. And we used the support vector machine with RBF kernel, MLP with two hidden layers for all cases. The random forest used in this paper is a standard random forest with 2000 trees. To be fair, we used five-fold cross-validation to assess the overall accuracy of the different methods. The experimental results of classification accuracies were shown in Table 3.

**Table 3:** Comparison of precision of KNN, SVM, MLP, RF, gcForest, DFNForest, and LACFNForest on the GBM dataset

Method	KNN	SVM	RF	MLP	gcForest	DFNForest	LACFNForest
Precision	0.684	0.632	0.658	0.684	0.737	0.816	0.844
Recall	0.684	0.658	0.684	0.719	0.781	0.816	0.875
F-1 Score	0.684	0.645	0.671	0.702	0.759	0.816	0.860

**Fig. 2:**
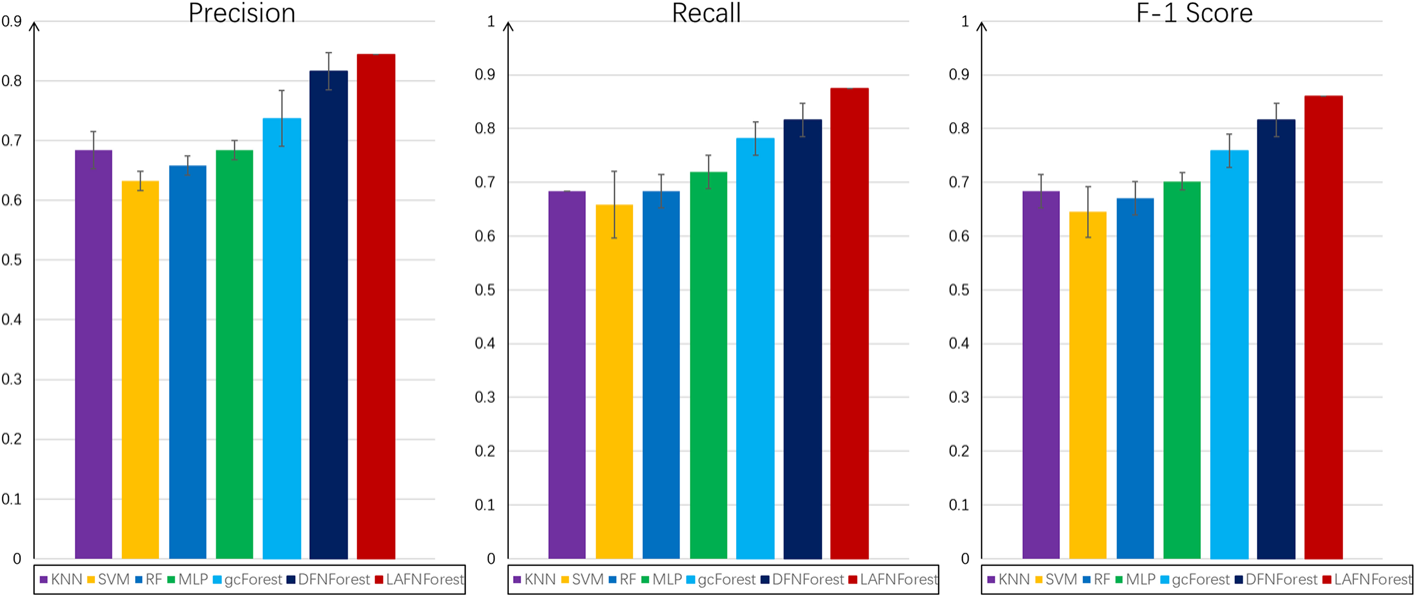
Comparison of the overall performance of multiple classifiers on the GBM dataset: average precision, recall, F-1 score and their respective deviation values

For the classification results of each classifier, we compared the average precision, recall and F-1 score, and give the deviation of the experimental results, as shown in Fig. 2.

It can be seen that LACFNForest achieves 84.4% in classification accuracy. Compared to other classifiers, LACFNForest still showed its superiority in classification performance on this dataset. At the same time, we can also notice that on the GBM dataset, the classification results are much lower than that on the BRCA dataset. This is due to the small number of samples in the training set. This occurrence confirms, to some extent, the problem we raised earlier. With the limited number of training samples that can be used, how to correctly classify each sample remains an issue that we need to further consider.

### Comparison between the LACFNForest and other classifiers on the LUNG dataset

The method combining fisher ratio and neighborhood rough set was used for gene selection on LUNG data set.We tested the performance of the proposed model for the classification of these cancer subtypes on the LUNG dataset. In the LUNG original dataset, each sample contains 4596 genes. The LUNG original data were used as the input for each classifier. For assessing the performance of LACFNForest and other classifiers, the five-fold cross-validation was performed in this data set. We let it be compared with KNN, SVM, MLP, RF, gcForest and DFNForest, respectively. The classification results of each classifier were compared in terms of average precision, recall and F-1 score, and the results are shown in Table 4.

**Table 4:** Comparison of precision of KNN, SVM, MLP, RF, gcForest, DFNForest, and LACFNForest on the LUNG dataset

Method	KNN	SVM	RF	MLP	gcForest	DFNForest	LACFNForest
Precision	0.710	0.776	0.746	0.791	0.830	0.865	0.880
Recall	0.746	0.791	0.746	0.776	0.791	0.830	0.873
F-1 Score	0.728	0.784	0.746	0.784	0.810	0.848	0.876

**Fig. 3:**
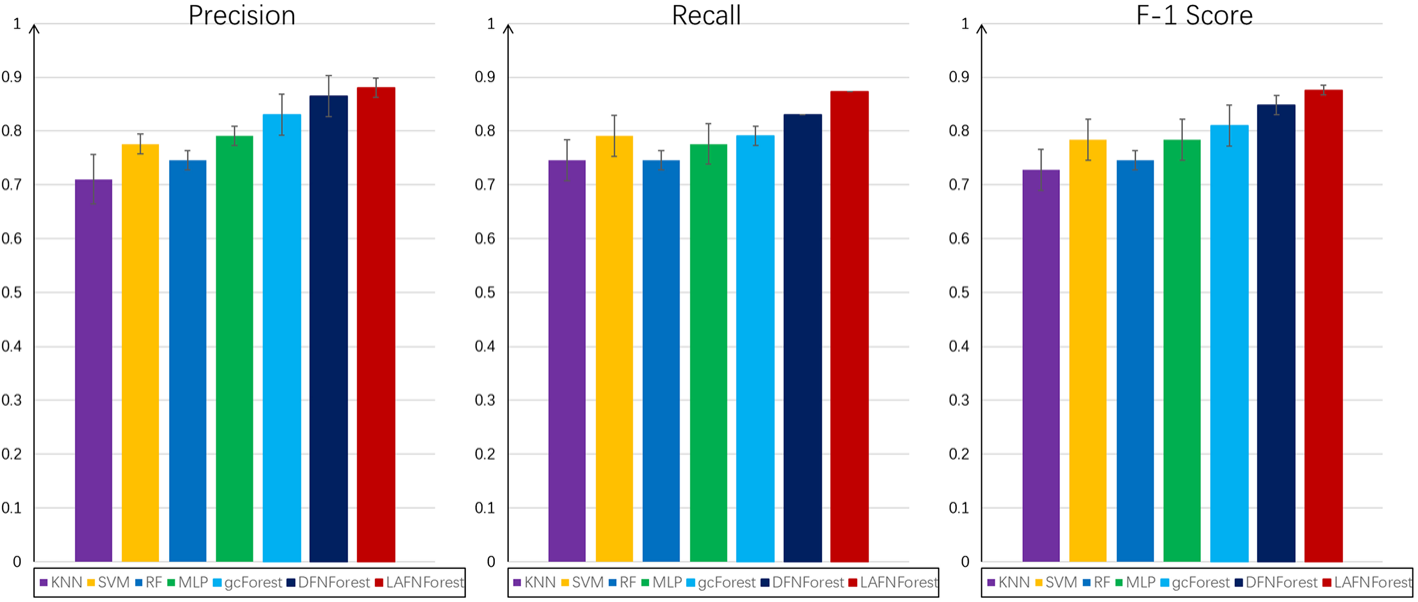
Comparison of the overall performance of multiple classifiers on the LUNG dataset: average precision, recall, F-1 score and their respective deviation values

In order to compare the classification results of different classifiers on the LUNG dataset more intuitively, we made the following comparison chart. As shown in Fig. 3.

**Fig. 4:**
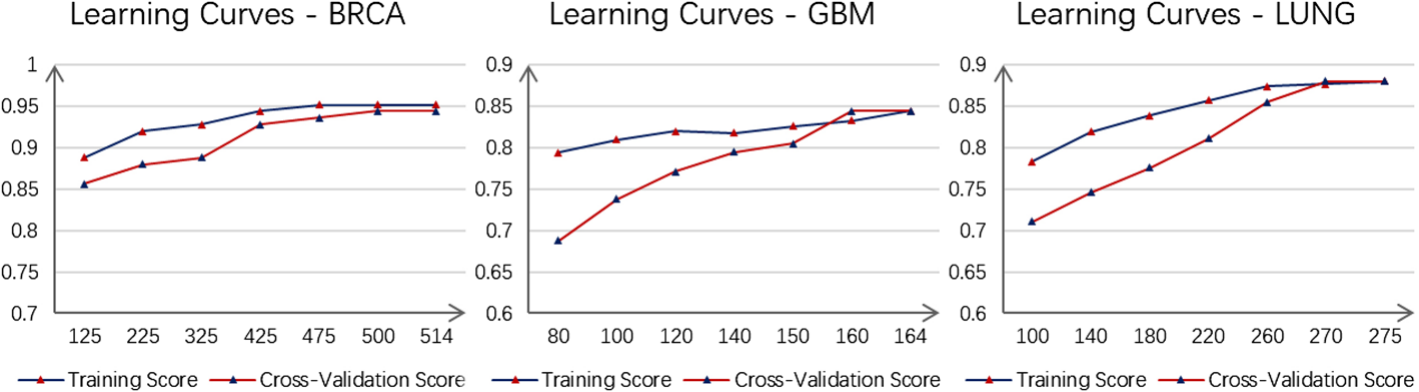
Learning curves of the laminar augmented cascading flexible neural forest model on BRCA, GBM, and LUNG datesets. The value in the abscissa of each coordinate system represents the number of samples used in the experiment. The ordinate value is the classification accuracy of the proposed model

As shown in Fig. 4, LACFNForest achieved a classification accuracy of 88% on the LUNG dataset, which was better than other classification models. Compared with KNN, SVM, MLP, RF and gcForest, the proposed model still showed better classification performance on this dataset. Experiments on the standard deviation of classification accuracy showed that the experimental results obtained by LACFNForest are more stable than other classification models.

### Over-fitting test

Determining whether a model is overfitted by producing learning curves is a common approach in the field of machine learning. From the learning curves, we can see how the model benefits as the number of samples increases, and determine what state the model is in. If the training score is very high but the cross-validation score is very low at the maximum sample size (i.e., the difference between the two curves is particularly large), then the model is generally considered to be in an over-fitting situation.

To test whether LACFNForest is over-fitting, numerous classification experiments were performed on each of the three data sets BRCA, GBM, and LUNG. For assessing the performance of the proposed model, five-fold cross-validation was performed on the proposed model. The original data were used as the input for each classifier. Learning curves on three data sets were made as shown in Fig. 4, and the classification accuracy was taken as the score of each experiment. Figure 4 showed the score of LACFNForest on the validation set(test set) and training set with the increase of the number of samples on three data sets.

As shown in the figure, all the learning curves showed an overall increasing trend as the number of samples increases, which means that with the increase of the number of samples available for the model, the performance of LACFNForest on both training set and testing set becomes better and better. Taking the learning curve on BRCA as an example, the training score has been in a relatively high position, while the cross-validation score has gradually increased to the highest classification accuracy of the model. When all the samples were used, the difference between the scores of the training set and the verification set is very small, which indicated that the proposed model is not over-fitting. On the GBM and LUNG data sets, although the score on the training set is relatively high at the beginning, the cross-validation score gradually increases. At the end, the difference between the scores of the training set and the verification set is very small, and the two curves almost coincide, indicating that the model is not over-fitted.

The main reasons why LACFNForest is not over-fitting are: (1) the base classifier FNT has sparse structure and supports cross-layer connection, which can effectively avoid over-fitting and has good generalization performance. (2) The model used DFNForest for each layer, and the number of layers in its cascade structure is determined automatically: when the accuracy on the validation set no longer increases, the number of levels will stop increasing.

## Discussion

When training neural networks, good classification results often rely on a large number of training samples [20]. However, it is difficult to find a model with high classification accuracy and good robustness when the number of training samples is small. Gene expression data of cancer subtypes were used as a continuous type of small sample size data. On the one hand, there is a strong correlation between gene expression information, which means that it is better not to discretize it. And this red will lead to many classifiers (e.g., decision trees, random forests) that tend to perform poorly when using gene expression data to classify cancer subtypes [27]. On the other hand, the high cost of acquiring gene expression data and the small number of samples available place demands on the structural and functional complexity of the classification model and on the adequate use of each sample by the classifier.

The number of classifiers and the diversity of classifiers tend to show a positive correlation with the performance of the ensemble model [28].However, too many classifiers and too complicated diversity not only bring a huge computation volume, but also cause a kind of “waste” to a large extent. How to make each classifier have enough “meaning” and interpretability while taking advantage of the ensemble learning is a problem to be considered when designing classifier architecture.

The proposed LACFNForest model uses DFNForest as its base classifier, which is not only capable of adaptively generating good neural network structure and parameters, but also capable of performing various kinds of classification tasks.Through the forced deepening of the forest structure, the model ensures that it still has enough structural complexity when dealing with small sample size data sets. We extend the deep neural forest to the densely connected deep neural forest, which further improves the prediction accuracy of the model. We introduce the output judgment mechanism to each layer of the forest to reduce the computational complexity of the model. This model also realizes the differential processing of different samples by judging the output of forest single-layer classification results, which reduces the computational complexity of the model. With the idea of layer-by-layer broadening model, the classification performance, diversity and robustness of the model have been further improved. LACFNForest provides a new way of thinking for the ensemble learning of the classifier in the structural design.

## Conclusion

Cancer is characterized by many subtypes, complex pathogenesis, and the possibility of mutation at any time. Correctly classifying the subtypes of cancer plays an extremely important role in promoting in-depth research on cancer in the medical field and realizing precise treatment for patients.

The close association between gene expression data and cancer subtypes makes the use of gene expression data for cancer subtype classification a natural advantage. However, the small sample size of gene expression data and the close association between genetic information make it difficult for many classifier models to classify them into subtypes. We proposed a laminar augmented cascading flexible forest model. The forced deepening of DFNForest ensures that the model was sufficiently complex to handle small sample size data sets and solves the problem of the limited number of FNTs in DFNForest. The accuracy of the model’s classification of each sample was ensured by introducing the structure of densely connected CNN into LACFNForest and assigning different weights to the classification results for each layer of forest. In addition, the hierarchical filtering of the sample points was used to prevent the model from being overly complex due to the increase in structural complexity.

More importantly, the idea of incremental broadening of the model was proposed, which not only strengthened the diversity and complexity of the ensembled model, but also avoided meaningless calculations as far as possible. The design gave the model better robustness and provided a new approach to the structural design of the classifier for ensemble learning. The experimental results showed that LACFNForest consistently outperforms state-of-the-art methods for classifying cancer subtypes using RNA-seq gene expression data.

## Methods

### Flexible neural tree

The advantages of artificial neural networks (ANNs) such as layer-by-layer processing, back propagation, and sufficient model complexity have made them a great success in all aspects of machine learning [29]. The excellent performance of deep learning built on various neural network models in areas such as classification and prediction has led to the further development of ANNs in terms of applications and algorithms.

**Fig. 5:**
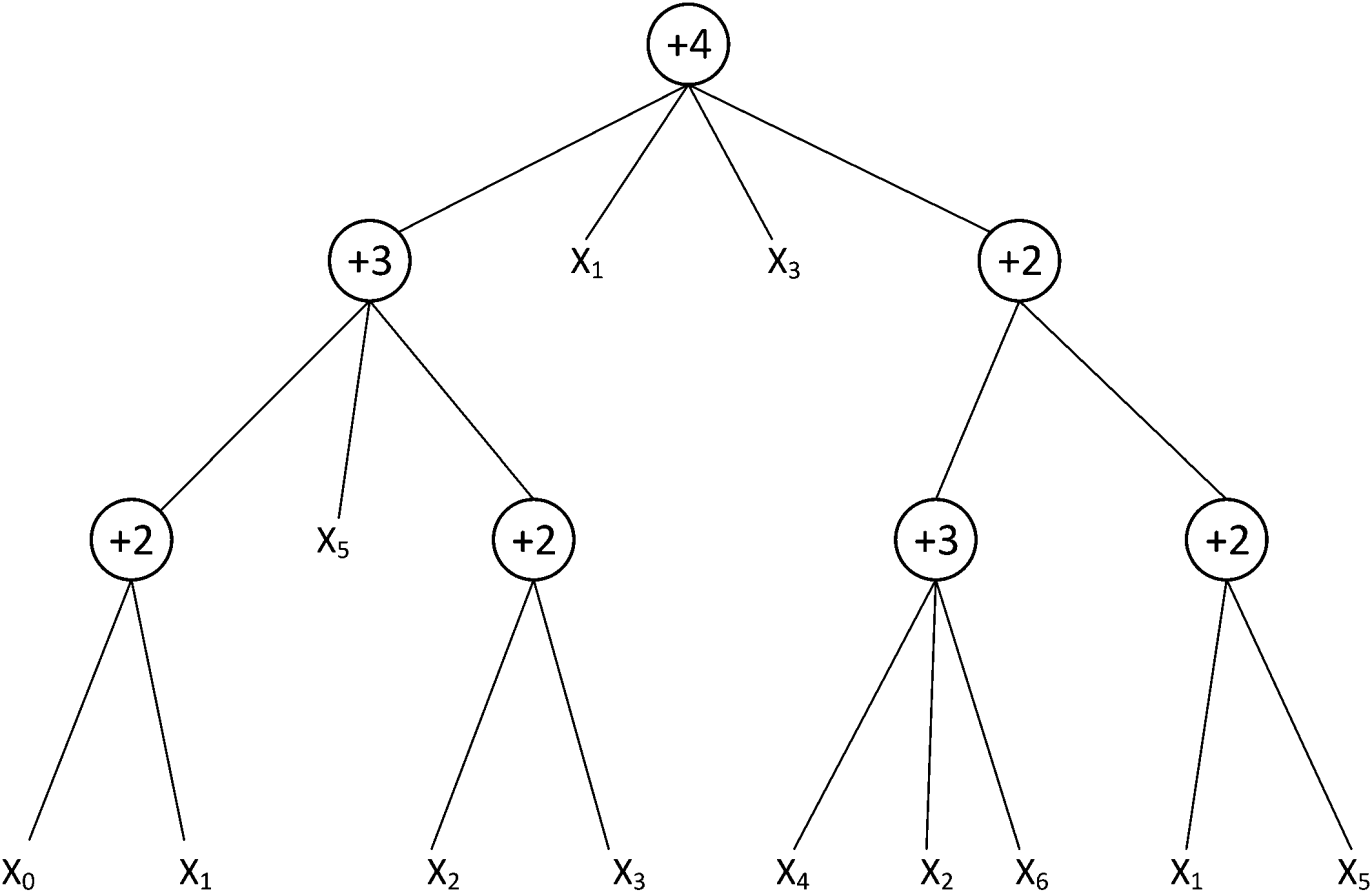
A typical representation of neural tree with function instruction set $F=\{+2,+3$, $+4,\},$ and terminal instruction set $\mathrm {T}=\left\{ \mathrm {x}_{0}, \mathrm {x}_{1}, \ldots , \mathrm {x}_{6}\right\}$

However, excessive hyper-parameter settings and the dependence of neural network structures on experience are persistent problems in the construction of neural networks [30]. Based on this, Chen et al. [31] proposed the flexible neural tree (FNT). The advantage of this model over other forms of artificial neural networks is that the FNT can adaptively determine its own structure during the training process, and the user does not need to give these hyperparameters in advance. And FNT can also adaptively select key features from all input features. It is worth mentioning that FNT allows cross-layer connection between nodes and input vectors, which can easily produce some network structures with sparse connections between nodes in adjacent layers (as shown in Figure 5). This is difficult to do when relying only on human experience to set the network structure [32].

To construct the FNT model, we choose the functional set (non-leaf nodes) $F=${+2,+3,+4} and the terminal instruction set (leaf nodes) $T=\left\{ x_{0}, x_{1}, \ldots , x_{6}\right\}$. The model is defined as follows

2 $$\begin{aligned}&S=F \cup T=\{+2,+3,+4\} \cup \left\{ x_{0}, x_{1}, \ldots , x_{6}\right\} \end{aligned}$$

3 $$\begin{aligned}&y_{\text{ non-leaf }}=\sigma \left( \sum _{j=0}^{M} \omega _{j} I_{j}+\theta \right) \ \end{aligned}$$

We can construct a structurally diverse tree neural network model by setting different functional sets for FNT. To increase the diversity of classifiers, three other structures with different function sets are used in LACFNForest:$F=\{+2,+3,+5\}, F 2=\{+2,+4,+5\}, F 3=\{+3,+4,+5\}$.The algorithmic rules for the FNT model are shown in Table 5.

**Table 5:** Algorithmic rules for flexible neural tree models

1	The output of the leaf node is the value of a given input feature variable
2	The output of a flexible neuron $+M$(non-leaf nodes) can be produced as formula (3) where $I_j$ is the input to the current node, $\omega _j$ is the corresponding weight and $\theta$ is the node’s oset or bias
3	The output of each node is used as the input value of the node to which it is connected at the previous level
4	Calculate the value of the output vector from bottom to top, from leaf node to root node

**Fig. 6:**
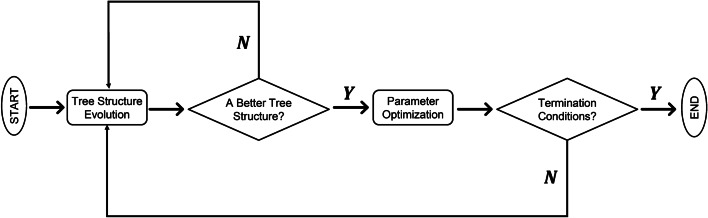
The pipeline of FNT. It mainly includes two parts: tree structure evolution process and parameter optimization process. The algorithm stops when the optimal tree structure is generated and the termination conditions are met

In terms of the choice of the activation function $\sigma ( x ),$ three common nonlinear activation functions are: Gaussian: $\sigma (x)=\exp \left( -\left( \frac{x-b}{a}\right) ^{2}\right) ,$ Logistic: $\sigma (x)=\frac{1}{1+\exp (-x)}$, ReLU: $\sigma (x)=\max (0, x)$. In this paper we use logistic function as the activation function for FNT [33]. The pipeline of FNT is shown in Fig. 6.

In the population generated during the optimization of the FNT structure and parameters,the individual is determined by the size of the adaptation function *Fit*(*i*). This paper uses standard variance as the adaptation function.

4 $$\begin{aligned} F i t(i)=\sqrt{\frac{1}{N} \Sigma \left( y_{1}^{i}-y_{2}^{i}\right) ^{2}} \end{aligned}$$

where *N* denotes the total number of samples. $y_{1}^{i}$ and $y_{2}^{i}$ are the actual value of the *i*-th sample and the output of the FNT. *Fit*(*i*) denotes the adaptation value of the *i*-th tree. *Fit*(*i*) reflects the error between the actual output and the expected output [34]. For a flexible neural tree, the smaller the value of *Fit*(*i*), the smaller the error between the actual output and the expected output, which means that the actual output is very close to the expected result. The smaller the value of *Fit*(*i*), the better FNT we will get [35].

### Deep flexible neural forest

**Fig. 7:**
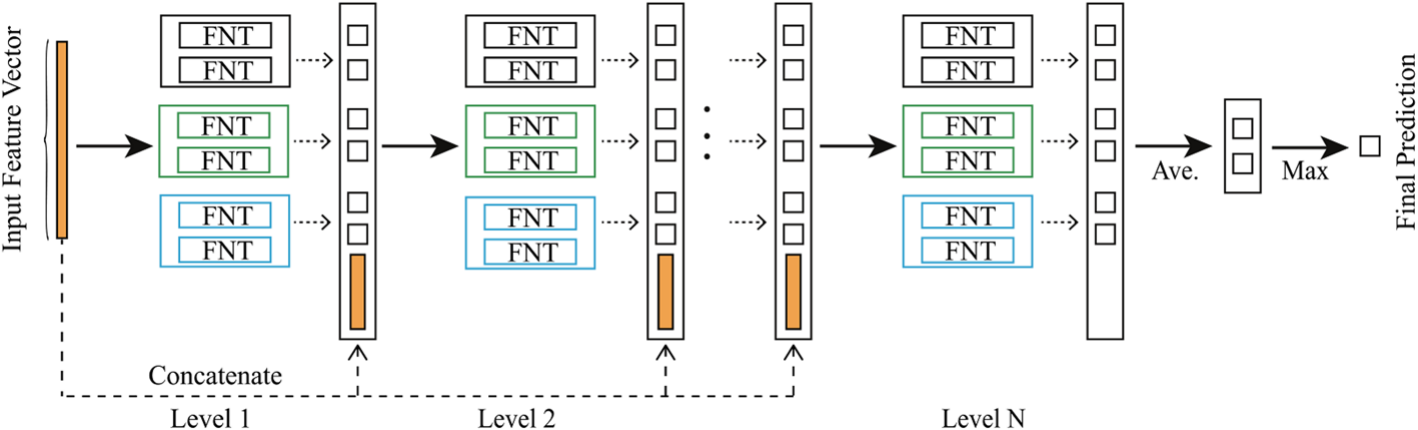
A deep flexible neural forest model. Each forest contains $\log _{2} M$ FNTs which are deepened by cascading. The output of the model is the average of all FNT outputs in the last level

Although FNT have many advantages that other artificial neural networks do not have, the single-output nature dictates that it cannot be used directly to deal with multi-classification problems. To solve this problem, Xu et al. [25] proposed the deep flexible neural forest(DFNForest) model. As shown in Fig. 7.

The model uses the M-ary method to transform the multi-class problem into several two-class problems in a forest. The number of FNTs used in the forest is determined based on the number of dichotomous problems generated. For example, when using DFNForest for a four-class problem, two FNTs are needed in each forest $\left( \log _{2} 4=2\right)$.

Another work did by Xu is to deepen the FNT.DFNForest use grammar guided genetic programming (GGGP) and particle swarm optimization (PSO) algorithm [36] to evolve the structure and optimize the parameters values of FNT [37]. After the final output of each FNT is obtained, this output is merged together with the initial feature vector as an augmented feature vector. And they are sent as a new input to the next layer of FNT forest adjacent to it for processing. The number of layers of the entire forest is determined until the classification accuracy obtained by the neighboring layer forest is no longer increased, which has the advantage of deepening the FNT while not adding new parameters. In addition, Xu also increases the number and diversity of forests by setting three different functional sets for the FNTs.

### Laminar augmented cascading flexible neural forest

Although the performance of DFNForest in classifying cancer subtypes is better than other models [25], it still has many shortcomings. Firstly, the number of FNTs in DFNForest is limited by the problem to be solved - it can only be $\log _{2} M$ (*M* is the number of classes in the dataset to be classified). This results in a very small number of neural trees in the model. Too few FNTs will lead to instability in the classification performance of the whole model, and good classification results depend only on the performance of a single tree. In other words, the robustness of DFNForest is poor. Although the model has attempted to improve the diversity of the model and increase the number of FNTs by replacing different sets of functions. However, the number of trees is still too small for an integrated model to take advantage of integrated learning.Moreover, replacing the function set also brings an increase in other parameters.

Secondly, although DFNForest has deepened FNT by cascading it. However, since FNT is a better neural network by itself, it is easy to have a problem when using this model for classification(especially when dealing with small sample datasets): there are too few layers of model deepening due to the insignificant performance improvement of two adjacent layers. Insufficient model depth will not serve the purpose of deepening the FNT model well enough. In addition to this, DFNForest adds a very small number of new enhanced sample points per layer (the same number as for FNT). The model’s use of these sample points is limited to combining them with the original data samples and feeding them into the next layer. This results in inadequate use of the augmented samples and poor interpretability of the model.

**Fig. 8:**
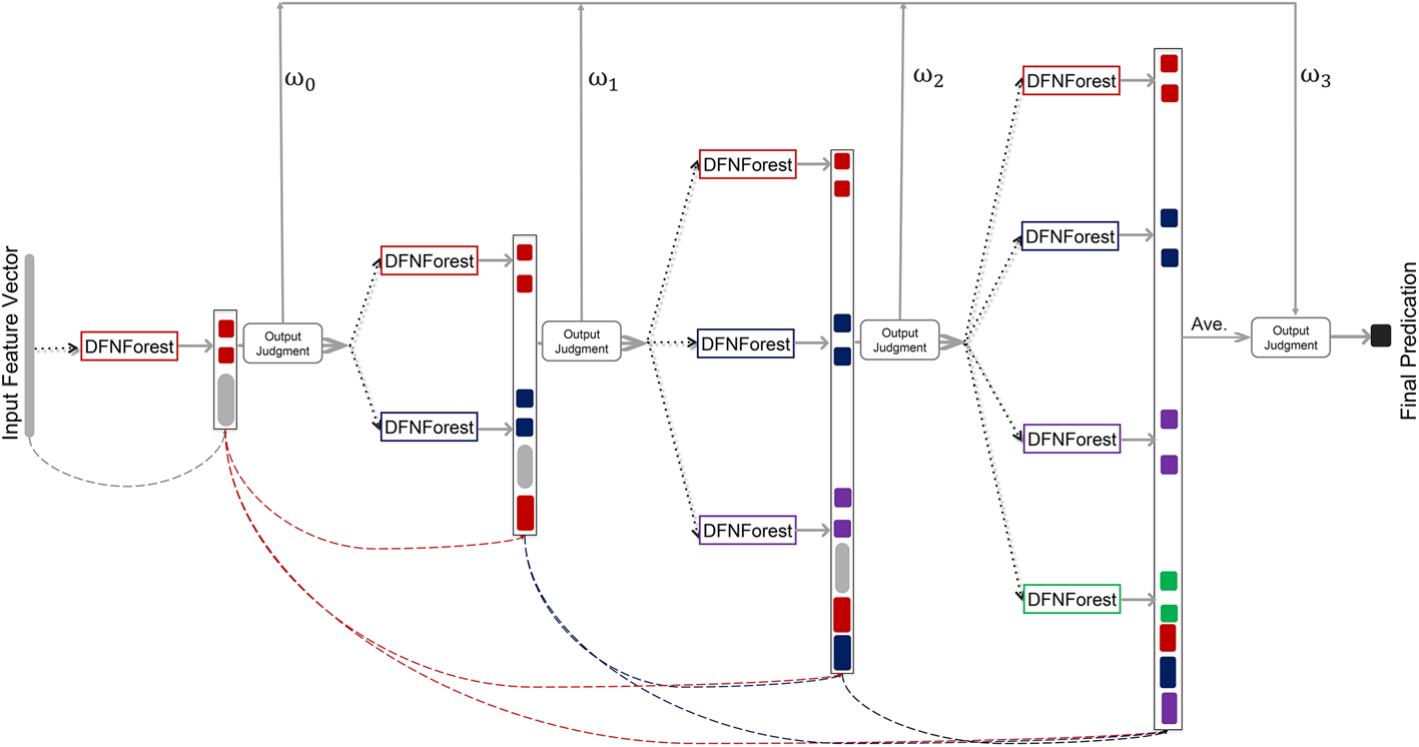
Laminar augmented cascading flexible neural forest model. We generate four forests with different syntaxes: the red forest uses the function set $\{+2,+3,+4\}$; the blue forest uses the function set $\{+2,+3,+5\}$; the purple forest chooses the function set $\{+2,+4,+5\}$ and the green forest uses the function set $\{+3,+4,+5\}$

Finally, DFNForest acts as a complex neural network computational model. Its computational complexity in calculating the output of each sample point is high. How to reduce the computational complexity as much as possible is also a problem to be considered. For these reasons, we propose a laminar augmented cascading flexible neural forest model. As shown in Fig. 8.

**Fig. 9:**
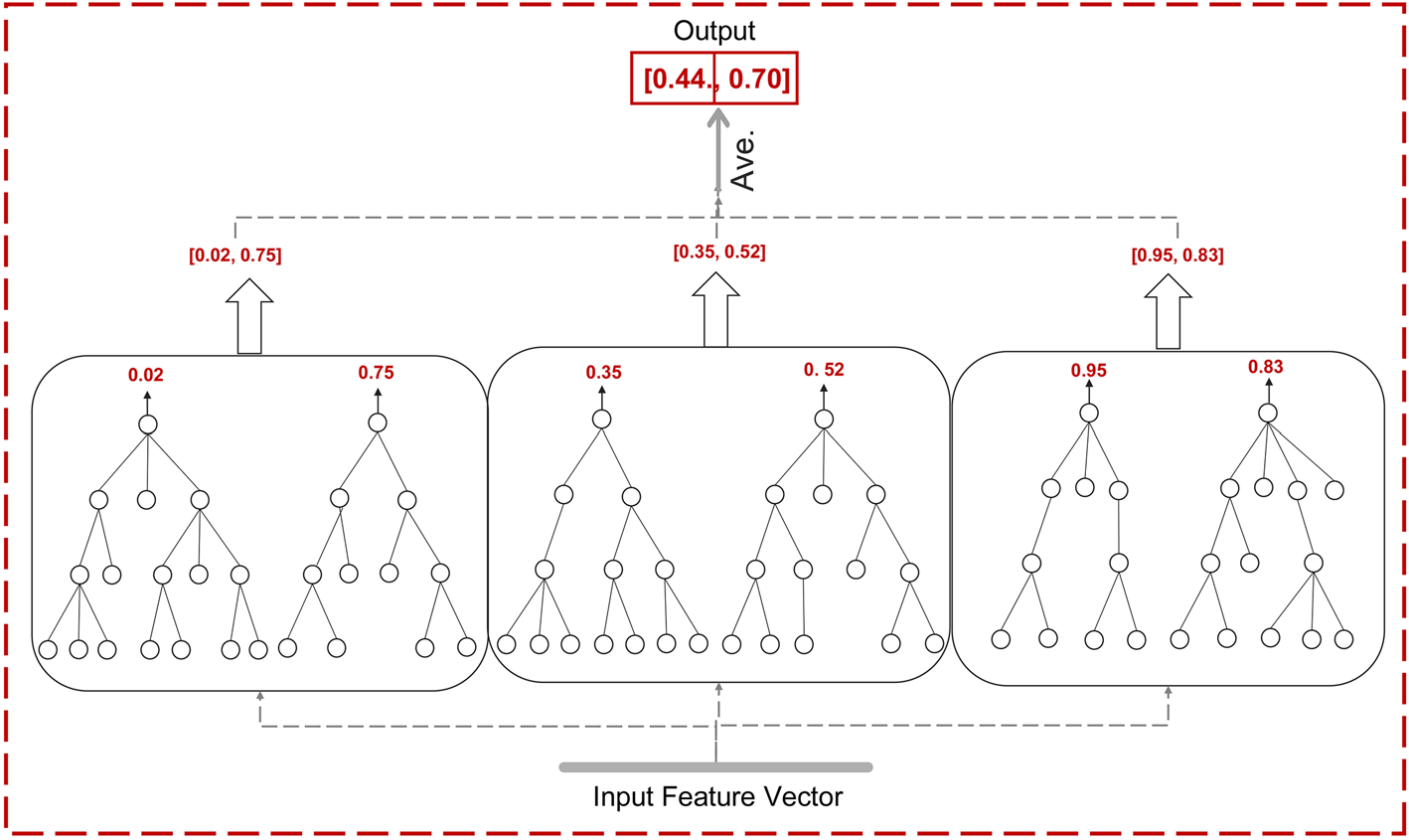
Forest first layer structure diagram. There are three DFNForests in this framework. For the four-class problem, there are two FNTs in each DFNForest. The two FNTs perform different two-class tasks respectively

We treat DFNForest as a base classifier capable of performing multiple classification tasks. The number in each DFNForest is $\log _{2} M * K$(*M* is the number of categories, and the value of *K* can be changed, $K \in N+$ ). For example, when dealing with the 4 -class problem, we assign *K* a value of 3, and the structure of the first layer of the model is shown in Fig. 9.

To prevent the forest deepening process from ending prematurely, we give the model a depth of 4. In this way, LACFNForest causes the FNT to be further forced to deepen to four times the original depth on top of the already deepened structure. As shown in Fig. 8, the output of the single-layer forest is the average prediction obtained after the *K* DFNForests stop deepening. This means that The convergence of the model is based on the performance of DFNForest. For each DFNForest, the training set is divided into two parts: one for training and the other for validation. When a new layer is added, the validation set verifies the entire cascade. The number of layers stops increasing when the classification accuracy stops increasing. In this way, the number of cascade layers can be determined automatically. After the number of all DFNForest layers within the fourth layer is determined, the structure of the entire model is determined.

After the processing of all the samples in each layer of the forest, we introduce an output judgment mechanism. We set a prediction confidence and divide the samples into two parts [38], one is the samples that have reached a high classification accuracy, which we call sample set *Y*. The other is the samples that have a low classification accuracy, which we call sample set *X*. For example, we give the prediction confidence interval as $[0 \sim 0.1] \cup [0.9 \sim 1] .$ Suppose there are 5 samples that have been processed in the first layer and the outputs are $\{0.07,0.35,0.52,0.83,0.95\}$. Then we can split the sample set into two parts: the two samples$\{0.07,0.95\}$ are within the prediction confidence interval we set, so these two samples belong to sample set *Y*. The other three samples that do not meet the confidence requirement belong to sample set *X*.

For each sample in *Y*, we have sufficient confidence in which class they can be classified without having to continue to send them into the next layer to be reclassified, while for each sample in the sample set *X*, the neural network structure and parameters constructed in all the previous layers have no way to classify them effectively, so we need to continue to optimize the model structure to continue to classify them. We deepened the entire model in a cascading way, while broadening the model structure by introducing a forest of different function sets, which increase the diversity of the forest on the one hand, and make the model can be adapted to deal with each sample by constantly building new neural network structures on the other.

The main purpose of widening the forest layer by layer and setting an output judgment mechanism is to solve the problem of excessively complex model calculations due to meaningless calculations. As the number of forest layers increases, the number of samples that meet the confidence level requirement increases layer by layer. At the same time, the number of samples that need to be sent to the next layer to continue the classification becomes smaller and smaller. Since we set the number of forest layers to 4, we change the prediction confidence to 0.5 after getting the output of the fourth forest layer to achieve the classification of all samples.

When calculating the final output of the sample, we wanted to avoid classification errors due to the large discrepancy between the classification results of a single layer of forests for the same sample and the classification results of other layers of forests. We chose to let the classification results of each layer participate in the final output of the sample with a certain weight. The result $y_{f}$ is calculated as

5 $$\begin{aligned} y_{f}=\sum _{i=0}^{N} \omega _{i+1} * y_{i+1} \quad \left( \omega _{i+1}=\frac{i+1}{1+2+\cdots +i+i+1}\right) \end{aligned}$$

where *N* is the number of layers, $y_{i}+1$ is the output of layer $i+1$ forest, and $\omega _{i+1}$ is the weight of the final output accounted for by the classification results of layer $(i+1)$-th forest.

**Fig. 10:**
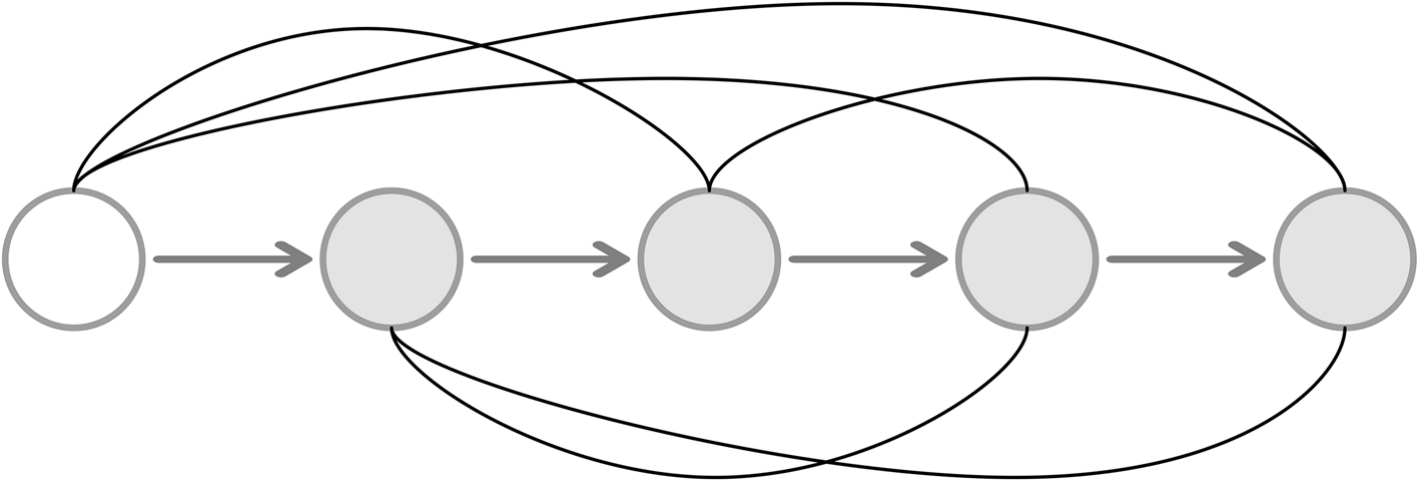
Dense block in densely connected CNN. The main idea of dense block is to add up all the outputs of prior layers as the input of subsequent neural layers

Finally, after processing the input samples at each layer of the forest, we were able to obtain $\log _{2} M * K$ augmentation samples. In using these augmented sample points, we refer to the fully connected CNN [39] structure, as shown in Fig. 10.

We let the augmented sample points of each layer be combined with the initial sample points to form a new input feature vector to be sent to the next layer of the forest for processing. On one hand, this can realize the full use of the augmented samples. On the other hand, as the number of forest layers increases, the number of augmented sample points increases. The densely connected use of augmented sample points ensures that the model still has a prominent performance advantage when dealing with small sample size datasets. This advantage will become more pronounced as the depth of the model increases.

## Data Availability

The gene expression data,miRNA expression data can be downloaded from The Cancer Genome Atlas web site at https://www.cancer.gov/about-nci/organization/ccg/research/structural-genomics/tcga. The specific BRCA, GBM and LUNG data sets in our manuscript were available through https://github.com/VeblenChung/Cancer-Subtype-Classification-Data-Set.

## Abbreviations

LACFNForest: Laminar augmented cascading flexible neural forest
BRCA: Breast invasive carcinoma
GBM: Glioblastoma multiforme
DFNForest: Deep flexible neural forest
DNN: Deep neural networks
DT: Decision tree
FNT: Flexible neural tree
gcForest: Multi-grained cascade forest
KNN: K-nearest neighbor
RF: Random forest
SVM: Support vector machine
TCGA: The cancer genome atlas
